# Detection of the endangered European weather loach (*Misgurnus fossilis*) via water and sediment samples: Testing multiple eDNA workflows

**DOI:** 10.1002/ece3.6540

**Published:** 2020-07-06

**Authors:** Lena Maureen Kusanke, Jörn Panteleit, Stefan Stoll, Egbert Korte, Eike Sünger, Ralf Schulz, Kathrin Theissinger

**Affiliations:** ^1^ Universität Koblenz‐Landau Landau Germany; ^2^ Umwelt‐Campus Birkenfeld Hoppstädten‐Weiersbach Germany; ^3^ Institut für Gewässer‐ und Auenökologie GbR Griesheim Germany; ^4^ Eusserthal Ecosystem Research Station University of Koblenz‐Landau Eusserthal Germany

**Keywords:** environmental DNA, freshwater fish, genetic monitoring, molecular protocols, monthly sampling

## Abstract

The European weather loach (*Misgurnus fossilis*) is classified as highly endangered in several countries of Central Europe. Populations of *M. fossilis* are predominantly found in ditches with low water levels and thick sludge layers and are thus hard to detect using conventional fishing methods. Therefore, environmental DNA (eDNA) monitoring appears particularly relevant for this species. In previous studies, *M. fossilis* was surveyed following eDNA water sampling protocols, which were not optimized for this species. Therefore, we created two full factorial study designs to test six different eDNA workflows for sediment samples and twelve different workflows for water samples. We used qPCR to compare the threshold cycle (*C*_t_) values of the different workflows, which indicate the target DNA amount in the sample, and spectrophotometry to quantify and compare the total DNA amount inside the samples. We analyzed 96 water samples and 48 sediment samples from a pond with a known population of *M. fossilis*. We tested several method combinations for long‐term sample preservation, DNA capture, and DNA extraction. Additionally, we analyzed the DNA yield of samples from a ditch with a natural *M. fossilis* population monthly over one year to determine the optimal sampling period. Our results showed that the long‐term water preservation method commonly used for eDNA surveys of *M. fossilis* did not lead to optimal DNA yields, and we present a valid long‐term sample preservation alternative. A cost‐efficient high salt DNA extraction led to the highest target DNA yields and can be used for sediment and water samples. Furthermore, we were able to show that in a natural habitat of *M. fossilis*, total and target eDNA were higher between June and September, which implies that this period is favorable for eDNA sampling. Our results will help to improve the reliability of future eDNA surveys of *M. fossilis*.

## Graphical abstract

The monitoring via environmental DNA (eDNA) is a useful tool to monitor threatened, hard‐to‐detect species such as the European weather loach (*Misgurnus fossilis*). To optimize the eDNA detection of this benthic fish species, we tested twelve different eDNA workflows for water samples and six workflows for sediment samples. Additionally, we monitored a ditch with an *M. fossilis* population over a year to determine the most suitable sampling period.

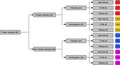

## 1 INTRODUCTION

Detecting an organism's DNA in the environment (eDNA) instead of the organism itself has been applied over the last decade in numerous studies concerning rare or invasive macroorganisms (Agersnap et al., 2017; Jerde, Mahon, Chadderton, & Lodge, 2011; Thomsen, Kielgast, Iversen, Wiuf, et al., 2012). The strong scientific interest is based on advantages eDNA has over traditional detection methods such as netting, traps, electrofishing, or visual surveys: it is noninvasive, more sensitive, less costly, and less time‐consuming (Lacoursière‐Roussel, Dubois, Normandeau, & Bernatchez, 2016; Lodge et al., 2012; Sigsgaard, Carl, Møller, & Thomsen, 2015). Commonly in the field of macrobial eDNA studies, single‐species approaches are used for conducting a presence/absence or quantitative monitoring of the target species (Barnes & Turner, 2016). The design of such aquatic eDNA monitoring studies raises many questions that must be answered in advance, concerning the choice of sample type, sample preservation, eDNA capture, eDNA extraction, and PCR assay. There are only a few published studies that conducted an in‐depth method testing for the eDNA detection of a target species (Brys et al., 2020; Deiner, Walser, Mächler, & Altermatt, 2015; Goldberg, Pilliod, Arkle, & Waits, 2011; Piggott, 2016). However, sampling and laboratory protocols adapted to the target organism are of high importance for successful detection and can significantly change the outcome of a monitoring study (Deiner et al., 2015; Hinlo, Gleeson, Lintermans, & Furlan, 2017).

*Misgurnus fossilis* has already been targeted in former eDNA studies (Brys et al., 2020; Sigsgaard et al., 2015; Thomsen, Kielgast, Iversen, Wiuf, et al., 2012). This species is especially interesting for eDNA monitoring because it spends most of its life hidden in the sediment and is classified as highly endangered in Germany and other countries in Central Europe (Federal Agency of Nature Conservation (BfN), 2008; Sigsgaard et al., 2015). Due to the drainage of swamps and a decreasing number of muddy backwaters and oxbows, this benthic fish species has suffered from severe habitat loss. Currently, agricultural ditch systems partly serve as replacement biotopes for *M. fossilis* populations. But even in such remaining suitable biotopes, the species has become rare in Central Europe, because it is affected by human interventions, for example, machine weeding or incautious dredging of sediment (Meyer & Hinrichs, 2000). To successfully apply conservation measures, it is important to identify existing populations to get an overview of the current distribution (Schreiber, Korte, Schmidt, & Schulz, 2018). However, *M. fossilis* is known to bury itself into the sediment and often occurs in habitats with periodically low water levels which is not favorable for traditional detection via electrofishing or fish traps (Meyer & Hinrichs, 2000; Sigsgaard et al., 2015). Thus, eDNA surveys appear to be a suitable alternative.

In the studies of Thomsen, Kielgast, Iversen, Wiuf, et al. (2012) and Sigsgaard et al. (2015), *M. fossilis* was surveyed following the eDNA water sampling protocol of Ficetola, Miaud, Pompanon, and Taberlet (2008), which was originally used for the detection of the American bullfrog (*Rana catesbeiana*). Furthermore, in both studies, commercial tissue kits were used for DNA extraction similar to the study of Ficetola et al. (2008). Although both studies successfully detected *M. fossilis* at certain sampling sites, it has not been tested whether the applied sampling and laboratory protocols provide an optimal DNA yield for *M. fossilis*. It has been shown that the efficiency of DNA extraction protocols varies greatly between organisms, due to their different physiological and chemical properties as well as the properties of their natural environments (Wang et al., 2013). An ineligible DNA extraction protocol can lead to false‐negative results and consequently to underestimation of certain species in a sample (Morgan, Darling, & Eisen, 2010; Wang et al., 2013). Furthermore, *M. fossilis* predominantly lives in the sludge at the bottom of almost stagnant water bodies (Meyer & Hinrichs, 2000), which could lead to an underrepresentation of its DNA in the water column. Thus, sediment samples might be more suitable for their detection than water samples (Turner, Uy, & Everhart, 2015). Recently, the study of Brys et al. (2020) has shown that also the choice of PCR method (quantitative PCR [qPCR] or digital droplet PCR [ddPCR]) and the choice of PCR assay is of great importance for the detection success of *M. fossilis*. In our study, we focused on the steps prior to PCR: sample type, sample preservation, DNA capture, and DNA extraction. We hypothesized that monitoring of *M. fossilis* via eDNA could be more reliable if both sample types (water and sediment) were collected and analyzed to minimize false‐negative results. Furthermore, it is important to consider that eDNA shedding, persistence, settling, and transport are strongly influencing a species’ detectability (Sansom & Sassoubre, 2017). These factors are reliant on many environmental parameters such as nutrition of the target organism, UV radiation, enzymatic activity, water temperature, and water quality, which vary considerably over the year (Eichmiller, Best, & Sorensen, 2016; Klymus, Richter, Chapman, & Paukert, 2015; Sansom & Sassoubre, 2017). Thus, it is important to determine a sampling period that is favorable for eDNA surveys.

In this study, we tested water and sediment samples with different workflows for long‐term sample preservation, eDNA capture, and DNA extraction to evaluate the target and total DNA yield (“methodical study”). We focused on long‐term sample preservation, which means a sample storage for at least 2 weeks, as it is not always possible to process a large number of samples within a short time as it recommended by Hinlo et al. (2017). We expected that the detection success varies significantly between water and sediment samples as well as between molecular workflows. Consequently, we hypothesized that the measured *C*_t_ values during the qPCR and the total DNA amount measured with the spectrophotometer will vary significantly between samples of different workflows. Because *C*_t_ values are inversely proportional to the target DNA amount (Heid, Stevens, Livak, & Williams, 1996), we used *C*_t_ values as an indication how much target DNA was contained in the samples. In the following, we use the term “target DNA yield/ amount” when we analyze the *C*_t_ values as the results of the qPCR. We use the terms “total DNA amount/ yield” to describe the DNA concentrations in ng/µl, which were measured in the DNA extracts using the spectrophotometer immediately after DNA extraction. Because environmental parameters can strongly fluctuate over the year in a natural habitat, we hypothesized that the detected eDNA yields will also vary over the year. We furthermore hypothesized that due to elevated activity during the breeding months of *M. fossilis* from April to June, the target eDNA yield in sediment and water samples will increase. Therefore, we took monthly water samples over one year at an agricultural ditch with a previously confirmed *M. fossilis* population. These water samples were subsequently analyzed regarding target and total eDNA yields to determine whether seasonal changes affect eDNA detection (“seasonal study”).

## 2 METHODS

### 2.1 Study sites

Environmental samples for the methodical study were taken from a fishpond (6.45 m × 16 m × 1 m) at the Eußerthal Ecosystem Research Station, Germany (49.2544, 7.9616), in which 40 *M. fossilis* were held in four cages (145 cm × 54 cm × 87 cm) over several months for a breeding program. The fishpond resembled the natural *M. fossilis* habitat with plenty of riparian plants, macrophytes, and a thick sediment layer. The bottoms of the cages were submerged approximately 12 cm into the sediment.

The samples for the seasonal study were taken from a ditch in Rheinzabern, Germany (49.09324, 8.30060). The study site was chosen because there was an *M. fossilis* population detected during past surveys (Korte, unpublished data, pers. comm., 2017). The ditch is in total 2.9 km long and connected to three other ditches in the area. At the sampling location, the ditch is 150 cm wide and 100 cm deep. The sludge layer is approximately 40 cm thick, and the water level is up to 60 cm. It has a narrow strip with riparian plants and plenty of macrophytes. The ditch is surrounded by agriculture and is close to a federal highway. One part of the ditch passes under the highway via a culvert. We took water samples monthly from May 2017 to April 2018, while in December and January, sampling was not possible due to dryness. The water samples were always taken at the same sampling point. In April 2018, nine days after the last eDNA sampling, we confirmed a population of at least 20 individuals using fish traps (Table S8). The total size of the population is yet unknown.

### 2.2 Study design

**FIGURE 1:**
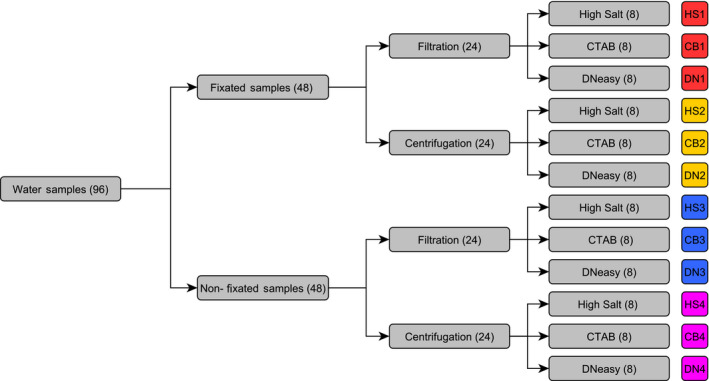
Experimental set‐up for water samples, testing the DNA yield of different combinations of sample preservation, DNA capture, and DNA extraction strategies in a full factorial design. Numbers in parenthesis represent the number of samples (N). An ID was assigned to each of the twelve workflows

**FIGURE 2:**
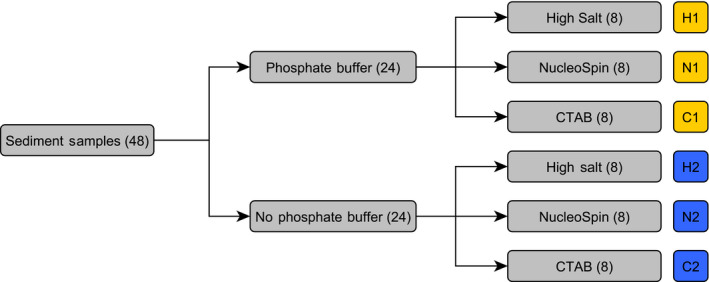
Experimental set‐up for sediment samples, testing the DNA yield of six different method combinations of sample pretreatment and DNA extraction using a full factorial design. Numbers in parenthesis represent the number of samples (*N*). An ID was assigned for each of the six workflows

For the methodical study, we developed two full factorial study designs, one for water samples (Figure 1) and one for sediment samples (Figure 2), to test the DNA yields of various workflows.

For the water sample experiment, 96 samples were taken in total (Figure 1). Of these 96 water samples, 48 samples contained 15 ml pond water and were fixated with ethanol and sodium acetate directly at the sampling site, following the method of Ficetola et al. (2008). The other 48 water samples were frozen and not fixated, and each sample contained 200 ml of pond water. Of the 48 fixated and nonfixated samples, respectively, 24 samples were filtered at the laboratory using cellulose nitrate filters with a pore size of 0.45 µm (Ø 47 mm, Sartorius, Göttingen, Germany), a glass vacuum filtration device (Ø 47 mm, DWK Life Sciences, Wertheim, Germany), and a water‐jet pump (ROTH SELECTION, Carl Roth GmbH + Co. KG, Karlsruhe, Germany). The remaining 24 samples were centrifuged for 35 min at 6°C to create an organic pellet for subsequent DNA extraction. After the centrifugation or filtration step, three different DNA extraction methods were tested (*N* = 8 each). The first DNA extraction method was a modified high salt protocol from Aljanabi and Martinez (1997). Second, we tested a CTAB (Cetrimonium bromide) DNA extraction protocol (Coyne, Hutchins, Hare, & Cary, 2001; Turner et al., 2015). As the third DNA extraction method, we used the DNeasy Blood & Tissue Kit (Qiagen GmbH, Hilden, Germany). The described study design resulted in 12 different workflows (*N* = 8 each; Figure 1).

For our sediment experiment, we took 48 sediment samples. We tested six different workflows (*N* = 8 each), including a sediment pretreatment versus no pretreatment and three different DNA extraction methods in a full factorial design (Figure 2). We used a phosphate buffer protocol developed for soil samples by Taberlet et al. (2012) as a sediment pretreatment (*N* = 24). For the other 24 samples, no sediment pretreatment was used before DNA extraction (Figure 2). The tested DNA extraction methods were: (a) a modified high salt protocol (Aljanabi & Martinez, 1997, modified), (b) a CTAB protocol (Coyne et al., 2001; Turner et al., 2015), and (c) the NucleoSpin Soil Kit (Macherey‐Nagel). The six different workflows were given a unique ID in the following chapters (Figure 2).

### 2.3 Decontamination

To avoid sample contamination with DNA, we used new, unused sampling bottles for all sediment and water samples. Disposable gloves were worn and regularly changed during the sampling and while working in the laboratory. Equipment such as tweezers, scissors, pipettes, and measuring spoons, that had direct contact with the samples, was cleansed with 10% bleach and rinsed with ultrapure water before each use and between each sample. To clean the filtration devices, we created a bleach bath containing 5 L of water and 200 ml of a cleaning agent containing bleach (DanKlorix, Hygiene‐Reiniger, CP GABA GmbH, Hamburg). Filtration devices were soaked in the bleach bath for 10 min and then rinsed with ultrapure water. After each cleaning of the filtration device, 200 ml of tap water was poured through a new filter as negative controls.

To avoid contamination in the laboratory, we used separate rooms for pre‐ and post‐PCR processes and filtration. All master mixes for the qPCR were prepared in a PCR UV cabinet (Captair Biocap DNA/RNA Workstation, Erlab, Köln). All sample containers that were brought from the field into the laboratory were cleaned from the outside with 10% bleach and rinsed with ultrapure water. Before and after each DNA extraction or qPCR, all relevant surfaces were cleaned with 10% bleach and then wiped with ultrapure water.

### 2.4 Water and sediment sampling

In total, we took 96 water samples from inside the cages in which the *M. fossilis* were held. The water samples were taken on the 12th of November 2018. Of the total 96 water samples, 48 samples were fixated directly at the sampling site, following Ficetola et al. (2008). Therefore, 33.5 ml of 100% ethanol and 1.5 ml of 3 M sodium acetate were added to 15 ml of pond water. Additionally, three negative controls were made using 15 ml tap water and fixating it as described above at the sampling site. The other 48 water samples each contained 200 ml pond water and were not fixated. The three negative controls contained 200 ml tap water, and the bottles were taken to the sampling site. All 96 water samples were stored on ice during the transport to the laboratory. The samples were stored at −20°C for at least two weeks, before DNA capture (filtration/ centrifugation) and then DNA extraction were conducted.

We took a total of 48 sediment samples on the 13th of February 2019 from the same pond where the water samples were taken. Before the sediment sampling started, we took one 1.5 L water sample for comparison. Superficial sediment was scraped by hand into 50 ml tubes from the bottom of the *M. fossilis* cages until the tubes were three‐quarter full. For the workflow C2 (Figure 2), we followed the sediment protocol of Turner et al. (2015) and added 5 ml of superficial sediment with a measuring spoon to a 50 ml centrifuge tube containing 10 ml of CTAB buffer per sample. As negative controls, three 50 ml tubes were filled with 10 ml CTAB buffer, using a clean 5 ml measuring spoon at the sampling site. All samples were preserved on ice during transport and stored at −20°C for at least two weeks at the laboratory.

For the seasonal study, we took one 1.5 L bottle of water from the ditch with the natural *M. fossilis* population monthly from May 2017 to April 2018. The bottles were transported on ice and stored at −20°C for several months at the laboratory.

### 2.5 DNA capture in water samples and sediment pretreatment

We compared centrifugation and filtration as two different DNA capture methods for water samples. Centrifugation was conducted in six of the twelve tested workflows, so in total, 48 samples were centrifuged (Figure 1). Of these 48 centrifuged samples, 24 samples were fixated and contained 15 ml pond water, 33.5 ml 100% ethanol, and 1.5 ml 3 M sodium acetate. The other 24 centrifuged samples were not fixated and contained 200 ml frozen pond water. We centrifuged all samples at 6°C for 35 min and subsequently discarded the liquid by pouring it off carefully. The organic pellet was then used for DNA extraction. The 200 ml samples were thawed in a water bath at 30°C and then split into four 50 ml tubes for centrifugation, and the organic pellets were pooled afterward for the DNA extraction. The fixated samples were not frozen because they contained ethanol and they were already in 50 ml centrifugation tubes, so they were centrifugated in their original sampling containers.

For six of the workflows, we used filtration as a DNA capture method (*N* = 48, Figure 1). The filtration was conducted in the laboratory using cellulose nitrate filters with a pore size of 0.45 µm, a glass filter holder, a filtration flask, and a water‐jet pump. Each filter was stored separately in a 2 ml tube at −20°C until the DNA extraction. The 1.5 L water samples for the seasonal study and the 1.5 L comparison water sample taken right before the sediment sampling were also filtered using cellulose nitrate filters with a pore size of 0.45 µm. We used two filters for the 1.5 L water sample that was taken at the fishpond before the sediment sampling, because the first filter was clogged after filtering approximately half of the sample volume. The filters were then treated as two separate subsamples.

The frozen sediment samples were thawed at 30°C in a water bath. In three out of six sediment workflows (*N* = 48), sediment samples were pretreated with a phosphate buffer protocol developed for soil samples by Taberlet et al. (2012, Figure 2). Following this protocol, we used a saturated phosphate buffer (Na_2_HPO_4;_ 0.12 M; pH ~ 8) to separate the DNA from the sediment. For the workflows including a phosphate buffer treatment and extracted with the high salt protocol (H1), the NucleoSpin Soil Kit (N1), and the CTAB extraction (C1), we used 20 g of sediment for one sample and added 20 g of phosphate buffer. The mixture was then incubated for 30 min at room temperature, while vortexing it every 5 min at the highest speed. Subsequently, a 1 ml aliquot of the sediment–buffer mixture was taken and centrifuged for 10 min at 10,000 ***g***. Afterward, 500–700 µl of the supernatant was taken and used for the subsequent DNA extraction method: 500 µl for NucleoSpin Kit (Macherey‐Nagel, Düren), 600 µl for high salt protocol, and 700 µl for CTAB protocol. We used different volumes for each extraction method because we maximized the amount of starting material for each workflow, while also considering the recommendations of the original protocols and technical constraints.

For the other three sediment workflows, no pretreatment with phosphate buffer was conducted (Figure 2). We used 500 mg of untreated sediment for the workflow N2 including the NucleoSpin Kit (Macherey‐Nagel, Düren) following the manufacturer's protocol. We also used 500 mg of untreated sediment for the workflow H2 including the high salt DNA extraction protocol. For workflow C2, we followed the protocol of Turner et al. (2015) and pretreated one 5 ml measuring spoon of sediment per sample with 10 ml of CTAB buffer directly at the sampling site. The sediment samples of workflow C2 were stored with the CTAB buffer at −20°C until DNA extraction.

### 2.6 DNA Extraction

#### 2.6.1 Water

For the water samples, we tested three different DNA extraction methods: (a) a high salt protocol (Aljanabi & Martinez, 1997; modified), (b) a CTAB extraction method (Coyne et al., 2001; Turner et al., 2015), (c) the Qiagen DNeasy Blood & Tissue Kit (Qiagen GmbH, Hilden, Germany). The high salt protocol used in this study was based on the protocol of Aljanabi and Martinez (1997), but was considerably modified for our purpose. A complete description of the used high salt protocol is attached in Appendix S2. The DNA pellets of the high salt protocol were resuspended in 100 µl H_2_O. The monthly water samples from the ditch were also extracted using the described high salt protocol for filter samples as well as the two filters from the comparison water sample that was taken before the sediment sampling. The two filters from the comparison water sample were extracted as two separate subsamples. For the CTAB method, we followed the water sample protocol described in the study of Turner et al. (2015) for centrifuged samples. For the filtered samples, we modified the protocol of Turner et al. (2015) by skipping the centrifugation steps and starting by incubating the filters in 700 µl CTAB buffer at 60°C for 10 min. The rest of the protocol was not modified. The DNA pellets of both centrifuged and filtered samples extracted with the CTAB method were resuspended in 100 µl of Low TE buffer. The DNA extraction for the centrifuged samples with the DNeasy Blood & Tissue Kit (Qiagen GmbH, Hilden) was conducted according to the manufacturer's protocol. For the filter samples fourfold, the amount of ATL buffer and proteinase K was used to cover the filters completely in liquid during the incubation. After the incubation, 200 µl of that mixture was used for the next steps of the protocol, following the manufacturer's instructions. The DNA for centrifuged and filtered samples extracted with the DNeasy Blood & Tissue Kit was eluted using 200 µl AE buffer. Two negative controls were used for each batch of DNA extraction.

#### 2.6.2 Sediment

We tested three different DNA extraction methods for the sediment samples (Figure 2): (a) a high salt protocol (Aljanabi & Martinez, 1997; modified), (b) a CTAB protocol (Coyne et al., 2001; Turner et al., 2015), (c) and the NucleoSpin Soil Kit (Macherey‐Nagel, Düren).

The high salt protocol was conducted as described in Appendix S2 for the water samples but using 500 mg of sediment instead of the water sample pellet (workflow H2, Figure 2). For the samples that were pretreated with phosphate buffer (workflow H1, Figure 2), the lysis step was skipped and 600 µl of the supernatant of the centrifuged phosphate–sediment mixture was used instead.

The CTAB protocol (Coyne et al., 2001; Turner et al., 2015) without phosphate buffer pretreatment (workflow C2, Figure 2) was conducted using the samples containing 5 ml of superficial sediment and 10 ml of CTAB and following the sediment extraction protocol described by Turner et al. (2015). For pretreated samples with phosphate buffer (workflow C1, Figure 2), 700 µl phosphate buffer mixture was added to 700 µl of Sevag buffer and then the above described CTAB protocol for water samples was used (Turner et al., 2015).

The DNA extraction with the NucleoSpin Soil Kit (Taberlet et al., 2012, Macherey‐Nagel, Düren) was performed using 500 mg of sediment and following the manufacturer's protocol (workflow N2, Figure 2). The lysis step was skipped while using the pretreated phosphate buffer samples (workflow N1, Figure 2). In this case, 500 µl of the sample–buffer mixture was used for the subsequent steps. For all sediment samples, except the samples extracted with the NucleoSpin Soil Kit (Macherey‐Nagel, Düren), 100 µl DNA extract was purified with the OneStep™ Inhibitor Removal Kit (Zymo Research, Irvine, CA). As the NucleoSpin Soil Kit (Macherey‐Nagel, Düren) already includes steps to remove inhibiting substances, it was not necessary to use the OneStep™ Inhibitor Removal Kit (Zymo Research, Irvine, CA) for those samples. Two negative controls were included for each batch of DNA extraction.

### 2.7 PCR and DNA quantification

A TaqMan qPCR assay previously developed for *M. fossilis* (Thomsen, Kielgast, Iversen, Wiuf, et al., 2012) was used to quantify the target DNA in the samples. The species‐specific primers and the probe for *M. fossilis* used in this study were the same as in Thomsen, Kielgast, Iversen, Wiuf, et al. (2012). The qPCR was performed using a Mastercycler RealPlex^4^ Epgradient S (Eppendorf, Hamburg) under the following conditions: 5 min 50°C; 10 min 95°C; 55x (30 s 95°C; 1 min 59°C). Fluorescence data collection by the FAM filter (520 nm) happened during the 59°C step. Duplicate PCR reactions were used with a total reaction volume of 10 µl. The PCR reactions each contained 6 µl Environmental Master Mix 2.0 (applied biosystems, Thermo Fisher Scientific, Woolston), 0.4 µl of each forward and reverse primer (10 µM), 0.4 µl of the TaqMan Probe (2.5 µM), 1.6 µl H_2_O, and 1.2 µl DNA extract. For each PCR run, we included two PCR negative controls, containing 1.2 µl water instead of 1.2 µl DNA extract. We also included a positive control for each PCR run, using a DNA extract from a tissue sample of *M. fossilis*.

Target DNA yield was analyzed using the comparative threshold cycle (*C*_t_) method. The *C*_t_ value is inversely proportional to the original abundance level of the target gene. It indicates the cycle number of the qPCR at which the measured fluorescence signal has crossed a fluorescence threshold. Thus, the *C*_t_ value is the PCR cycle number at which the exponential amplification of the target fragment has started (Heid et al., 1996). When no *C*_t_ value was measured during the entire PCR, and thus no DNA was present in the sample, we noted the *C*_t_ as 60, because our qPCR assay only included 55 cycles. We used two PCR replicates for the DNA samples extracted from the 96 water samples and 48 sediment samples of the methodical study. We used four PCR replicates for the DNA samples extracted from the monthly water samples of the seasonal study, because we only had one water sample of each month. We also used four PCR replicates for the DNA samples extracted from the 1.5 L water sample that we took before the sediment sampling at the fishpond.

The total eDNA concentration of the sediment and water samples was measured using the NanoDrop Spectrophotometer ND 1000. We measured each DNA sample five times with the NanoDrop Spectrophotometer and calculated the mean DNA concentration to compensate for the measuring error of this device.

### 2.8 Statistical evaluation

For the statistical analyses, we used R version 3.5.1 (© 2018 The R Foundation for Statistical Computing, Vienna, Austria). We used the packages car (Fox & Weisberg, 2011), carData (Fox, Weisberg, & Price, 2018), lawstat (Gastwirth, Hui, & Miao, 2019), and ggplot2 (Wickham, 2016). We tested whether the *C*_t_ values and the total DNA yields of each workflow were normally distributed using the Shapiro–Wilk test. We assessed the equality of variances of the *C*_t_ data and the total DNA data for the workflows that we wanted to compare using the Levene's test. To test whether there were significant differences between the total DNA concentration or the *C*_t_ values of the different workflows, we performed either a one‐way ANOVA (normal distribution and equality of variances) or a Kruskal–Wallis test. The pairwise Wilcoxon rank‐sum test was used as a post hoc test to compute pairwise comparisons between the workflows. As a *p*‐value adjustment method for multiple testing, we used the Benjamini–Hochberg (BH) method (Benjamini & Hochberg, 1995).

### 2.9 Calculation of detection rates

As we included eight sampling replicates for each workflow (Figure 1), and included two PCR replicates for each sampling replicate, this summed up to 16 PCR reactions in total per workflow. We calculated the percentage of positive PCR reactions of these 16 reactions for every workflow and referred to it as the detection rate.

## 3 RESULTS

All negative controls for sample collection, preservation, DNA extraction, and qPCR showed negative results, indicating that DNA contamination was successfully prevented.

### 3.1 Total DNA yield from water samples

**FIGURE 3:**
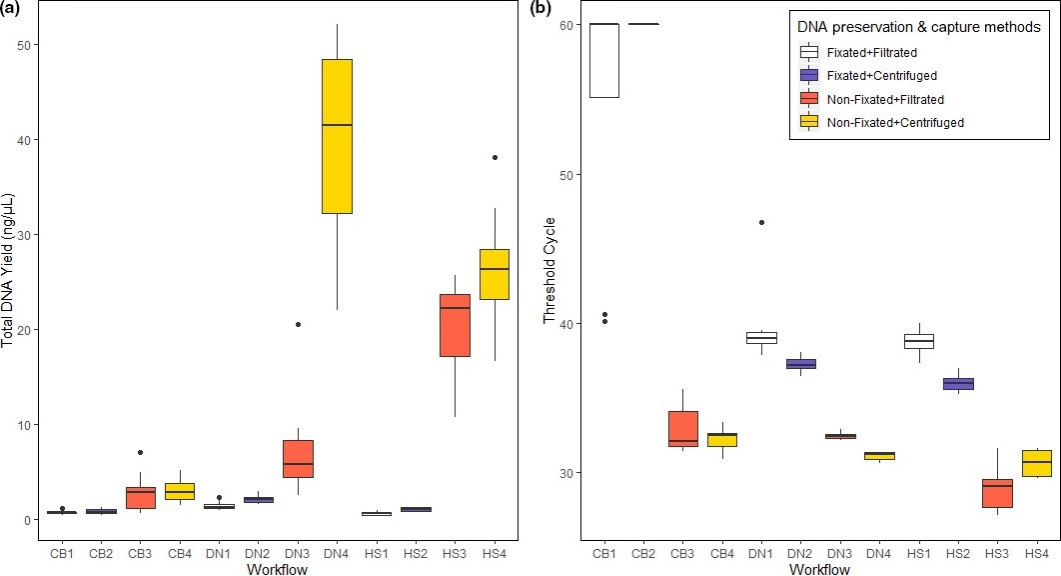
Total DNA yield (a) and threshold cycle values (b) measured in water samples of the twelve tested workflows (CB = CTAB DNA extraction, DN = DNeasy Blood&Tissue Kit, HS = high salt DNA extraction). *C*_t_ values are inversely proportional to the target DNA amount of a sample; thus, the lower the *C*_t_ value, the higher is the target DNA amount

The mean total DNA yield of the water samples ranged from 0.59 ng/µl found in workflow HS1 (fixation, filtration, high salt) to 39.4 ng/µl found in workflow DN4 (no fixation, centrifugation, DNeasy Kit; Figure 3). The total DNA yield of workflow DN4 was significantly higher than the yields of all other tested workflows (Table S1), however, with a standard deviation of 10.65. The workflow HS4 (no fixation, centrifugation, high salt) had the second‐highest total DNA yield of 26.21 ng/µl and a standard deviation of 6.65. The third‐highest total DNA yield of 20.25 ng/µl was achieved with workflow HS3 (no fixation, filtration, high salt; *SD* = 4.75). The overall comparison of the three different extraction methods showed that there were no significant differences in total DNA yield between samples extracted with the high salt DNA extraction protocol and the DNeasy Blood and Tissue Kit (*p*‐value = .17, Pairwise Wilcoxon rank‐sum test; Figure S1). However, samples extracted with the CTAB DNA extraction protocol had significantly lower total DNA yields than samples extracted with the two other extraction methods (high salt *p*‐value = .04, DNeasy *p*‐value < .001, Pairwise Wilcoxon rank‐sum test; Figure S1). Regarding sample preservation methods, nonfixated samples had significantly higher total DNA yields than fixated samples (*p*‐value < .001, Kruskal–Wallis test; Figure S2). Comparing the total DNA yields of the two DNA capture methods, centrifuged samples had significantly higher yields than filtered samples (*p*‐value = .04, Kruskal–Wallis test; Figure S3). The mean absorbance and mean ratio of absorbance at 260 and 280 nm, which were measured for the 12 different workflows for water samples, are listed in Table S2.

### 3.2 *C*_t_ values in water samples

The mean *C*_t_ values of the tested workflows ranged from 28.92 for workflow HS3 (not fixated filtered, high salt; Figure 3) to 60 for workflow CB2 (fixation, centrifugation, CTAB; Figure 3). The mean *C*_t_ value (*SD* = 1.39) of workflow HS3 was significantly lower than the *C*_t_ values of all other tested workflows (Pairwise Wilcoxon rank‐sum test; Table S5). The second‐lowest mean *C*_t_ value of 30.6 was obtained with workflow HS4 (*SD* = 0.81). The workflow DN4 lead to the third‐lowest mean *C*_t_ value of 31.08 (*SD* = 0.31; Figure 3). The overall comparison of the two sample preservation methods showed that nonfixated water samples had significantly lower *C*_t_ values than fixated samples (*p*‐value < .001, Kruskal–Wallis test; Figure S2). We found no significant difference comparing the *C*_t_ values between filtered and centrifuged samples (*p*‐value = .23, Kruskal–Wallis test; Figure S3). Comparing the *C*_t_ values of the three DNA extraction methods, we found that the high salt protocol had significantly lower *C*_t_ values than the CTAB protocol (*p*‐value = .003, Pairwise Wilcoxon rank‐sum test; Figure S1). There was no significant difference between the *C*_t_ values of samples extracted with the high salt protocol and samples extracted with the DNeasy Blood & Tissue Kit (*p*‐value = .07, Pairwise Wilcoxon rank‐sum test; Figure S1). Furthermore, there was no significant difference in *C*_t_ values of samples extracted with the CTAB method and samples extracted with the DNeasy Blood & Tissue Kit (*p*‐value = .03, Pairwise Wilcoxon rank‐sum test; Figure S1). The highest standard deviation of 8.51 was found among the samples of workflow CB1 (fixation, filtration, CTAB), with a mean *C*_t_ value of 55.08. In the samples of workflow CB2, no DNA was detected.

### 3.3 Total DNA yield from sediment samples

**FIGURE 4:**
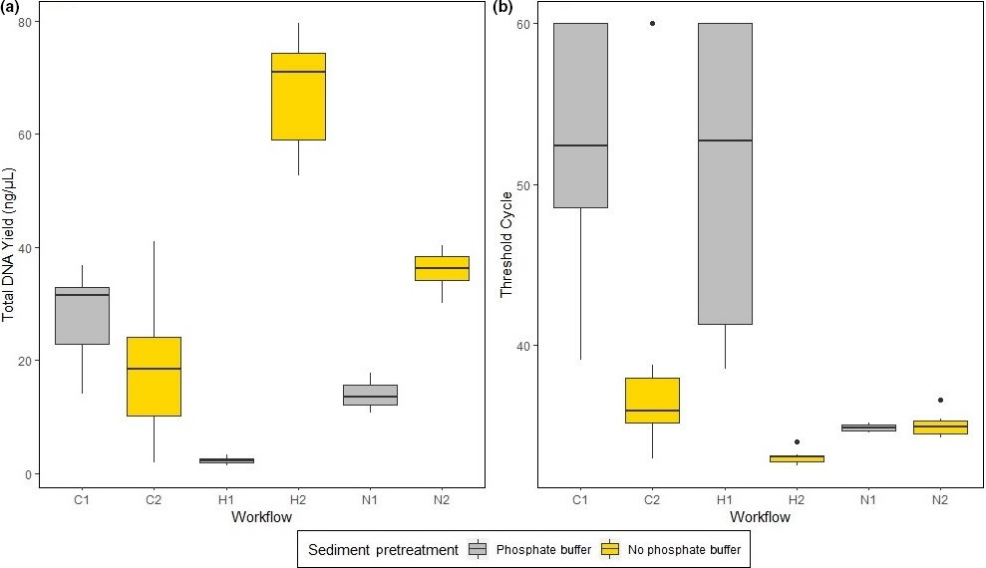
Total DNA yield (a) and threshold cycle values (b) measured in sediment samples of the six tested workflows (C = CTAB DNA extraction protocol, H = high salt DNA extraction, *N* = Nucleo Spin Soil Kit). *C*_t_ values are inversely proportional to the target DNA amount of a sample; thus, the lower the *C*_t_ value, the higher is the target DNA amount

The mean total DNA yield of the sediment samples ranged from 2.27 ng/µl in workflow H1 (phosphate buffer pretreatment, high Salt) to 67.89 ng/µl in workflow H2 (no pretreatment, high salt; Figure 4). The workflow H2 had a significantly higher mean total DNA yield than all other tested workflows (Table S3) and a standard deviation of 9.38. The second‐highest total DNA yield of 35.86 ng/µl (*SD* = 3.53) was obtained using workflow N2 (no pretreatment, NucleoSpin Kit; Figure 4). The third‐highest DNA yield with a mean of 28.19 ng/µl (*SD* = 7.1) was achieved following workflow C1 (phosphate buffer pretreatment, CTAB; Figure 4). The overall comparison of the total DNA yield in samples treated with phosphate buffer and without phosphate buffer showed that samples without phosphate buffer treatment had a significantly higher total DNA yield (*p*‐value < .001, Kruskal–Wallis test; Figure S4). Comparing the total DNA yield of samples treated with the three different DNA extraction methods, we found no significant differences that were caused only by the choice of the DNA extraction method (*p*‐value = .97, Kruskal–Wallis test; Figure S5). The standard deviations within the tested workflows were in most cases > 1, and only in workflow H1, a standard deviation < 1 was found (*SD* = 0.62). The highest standard deviation of 12.18 and a mean total DNA yield of 19.04 ng/µl were found for workflow C2 (no pretreatment, CTAB; Figure 4). The means of the absorbance and the ratio of absorbance at 260 nm/280 nm were calculated for each sediment workflow and listed (Table S4).

### 3.4 *C*_t_ values in sediment samples

The mean *C*_t_ values of the sediment samples ranged from 33.06 in workflow H2 (no pretreatment, high salt; *SD* = 0.43) to 52.37 in workflow C1 (pretreatment, CTAB; *SD* = 7.13, Figure 4). The workflow H2 had a significantly lower mean *C*_t_ value than all other workflows (Table S6). The second‐lowest mean *C*_t_ value of 34.85 and the lowest standard deviation of 0.22 were obtained using workflow N1 (phosphate buffer pretreatment, NucleoSpin Kit; Figure 4). The workflow N2 showed the third‐lowest mean *C*_t_ value of 35.04 (*SD* = 0.7, [Figure 4]). The highest standard deviation was found using workflow H1 (*SD* = 9.37, *C*_t_ = 50.79). The comparison of samples treated with and without phosphate buffer showed that samples without phosphate buffer had significantly lower *C*_t_ values (*p*‐value < .001, Kruskal–Wallis test; Figure S4). Comparing the three extraction methods, the NucleoSpin Soil Kit samples had significantly lower *C*_t_ values than the CTAB samples (*p*‐value < .001, Pairwise Wilcoxon rank‐sum test, Figure S5). There were no significant differences between the *C*_t_ values of samples extracted with the high salt protocol and the CTAB protocol (*p*‐value = .33, Pairwise Wilcoxon rank‐sum test, Figure S5) or between the high salt extraction and the NucleoSpin Kit (*p*‐value = .999, Pairwise Wilcoxon rank‐sum test; Figure S5). In the 1.5 L water sample (filtered, high salt) that was taken just before the sediment sampling as a direct comparison to the sediment samples, a mean *C*_t_ value of 33.95 was measured. Thus, the mean *C*_t_ value of this 1.5 L water sample was higher than the mean *C*_t_ value of workflow H2 (500 mg sediment, no pretreatment, high salt).

### 3.5 Detection rates

**FIGURE 5:**
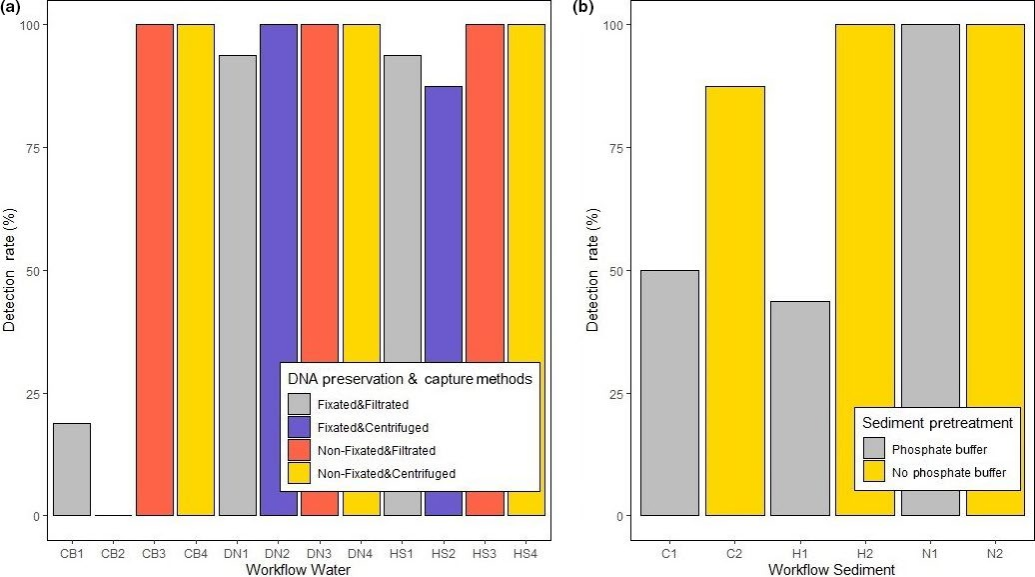
Detection rates of the twelve tested workflows for water samples (a) and sediment samples (b), shown is the percentage of positive samples among the 16 PCR reactions of each workflow (CB/ C = CTAB DNA extraction, DN = DNeasy Blood&Tissue Kit, HS/ H = high salt DNA extraction, *N* = Nucleo Spin Soil Kit)

Regarding water samples, we achieved a 100% detection rate in seven out of twelve tested workflows (Figure 5). All three extraction methods lead to a 100% detection rate when samples were not fixated and combined with either filtration or centrifugation. Additionally, a detection rate of 100% was achieved using workflow DN2 (fixation, centrifugation, DNeasy Kit; Figure 5). The workflow CB2 (fixated, centrifugated, CTAB) lead to a 0% detection rate, while with the workflow CB1 (fixated, filtered, CTAB) a detection rate of 18.75% was obtained. Regarding the sediment workflows, three out of six workflows achieved a detection rate of 100% (Figure 5). Both workflows including the NucleoSpin Soil Kit (N1, N2) and the workflow H2 (no phosphate buffer, high salt) lead to a detection rate of 100%. The lowest detection rate of 43.75% was achieved using workflow H1 (phosphate buffer, high salt; Figure 5). The workflow C1 (phosphate buffer, CTAB) had a detection rate of 50%, while workflow C2 (no phosphate buffer, CTAB) lead to a detection rate of 87.5%.

### 3.6 Total DNA yield and *C*_t_ values of monthly samples

**FIGURE 6:**
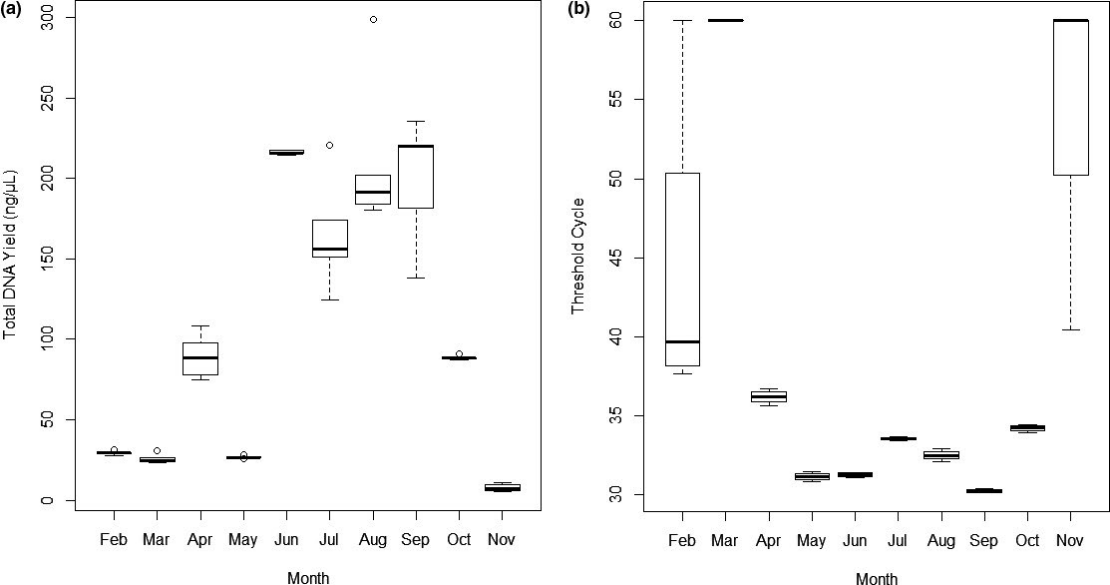
Total DNA yield (a) and threshold cycle values (b) measured in water samples from the surveyed ditch in Rheinzabern, Germany, for ten months of the year 2018 (n per month = 1, Nanodrop measurements per sample *n* = 4). January and December are missing because the ditch dried out. *C*_t_ values are inversely proportional to the target DNA amount of a sample; thus, the lower the *C*_t_ value, the higher is the target DNA amount

The total DNA yield was highest from June (mean = 216.08 ng/µl) to September (Figure 6). In November, February, and March, total DNA yields ranging from means of 7.92 ng/µl to 29.41 ng/µl were measured. In April, a peak in total DNA was measured, while in May, the total DNA was lower again. The qPCR analysis of the monthly water samples showed that target DNA concentration was higher from May to September than from October to April (Figure 6). The lowest *C*_t_ values and thus the highest target DNA concentration were measured in September (mean = 30.42). Out of the four qPCR replicates that were measured, the samples taken in February and November showed the highest standard deviation. The high standard deviation in these two cases occurred because not in all qPCR replicates DNA was successfully detected. In one of the February replicates and in three of the November replicates, no DNA was detected. Furthermore, all March replicates showed a negative result. In the replicates from May to October, the standard deviation was < 1. The mean values of the absorbance and the ratio of absorbance at 260 nm/280 nm measured for the samples of each month are listed in Table S7.

## 4 DISCUSSION

### 4.1 Water samples

In this study, we have shown that the long‐term preservation method of water samples has a significant effect on the total DNA yield and the target DNA yield. The fixation of water samples with ethanol and sodium acetate has been a common practice in previous eDNA studies targeting *M. fossilis* and other aquatic species (Ficetola et al., 2008; Sigsgaard et al., 2015; Thomsen, Kielgast, Iversen, Wiuf, et al., 2012; Turner et al., 2015). In this study, we have demonstrated that fixated water samples lead to significantly lower total and target DNA yields (Figure 3). The detection rates of fixated water samples were in five out of six tested workflows lower than the detection rates of nonfixated samples (Figure 5). It is feasible to argue that the lower total and target DNA yields in fixated samples are caused by the smaller sample volume (15 ml) compared to nonfixated samples (200 ml), rather than by the fixation method itself. The study of Turner et al. (2014) already postulated that the filtration of larger sample volumes maximizes the detection probability in surveys of rare organisms in large aquatic habitats. Contrary to this, Piaggio et al. (2014) found that the precipitation of samples fixated with ethanol and sodium acetate (15 ml water) performed better than a filtration protocol (2 L water) even though the fixated samples had a smaller water volume. It is not clear whether the small sample volume or the preservation method itself or a mixture of both factors lead to the significantly smaller DNA yields. However, we do not recommend using this preservation method with larger water volumes as it is impractical and wasteful to fixate large volumes of water. The here tested eDNA sample fixation method using ethanol and sodium acetate does not appear to be recommendable for future surveys of *M. fossilis* or similar monitoring programs at least for long‐term storage. Chemical fixation of water samples can be avoided by transporting water samples on ice and storing them at −20°C in a freezer, or by the direct filtration in the laboratory after sampling. In the study of Hinlo et al. (2017), it was shown that filtration within 24 hr after sampling leads to the highest DNA yield in water samples and that long‐term storage leads to a significant loss of DNA in the samples. Nevertheless, our study is focused on long‐term storage, because when large monitoring is done, oftentimes longer storage of samples cannot be avoided. There are many studies in which the water is filtered directly at the sampling site (Majaneva et al., 2018; Yamanaka et al., 2016), which is a good way to avoid long‐term storage. However, on‐site filtration is likely to be more prone to DNA cross‐contamination because it is not done in a controlled environment. Moreover, when the sampling is done by several different people, which is usual for larger monitoring programs, it takes less equipment and less expertise to simply take the water samples and store them. If long‐term storage is needed or not must be answered individually for every study. Yet, in this study, we were able to show that the choice of long‐term preservation method can change the outcome of a monitoring study significantly.

Regarding eDNA capture methods, we have demonstrated that centrifuged samples had significantly higher total DNA yields than filtered samples. This is inconsistent with the results of the study of Hinlo et al. (2017), in which higher DNA yields were obtained with filtration than with precipitation while using 250 ml water for filtration and 15 ml for precipitation. This inconsistency likely occurred because we used equal volumes of water for precipitation and filtration (for each method: 24 × 15 ml and 24 × 200 ml). Previous studies have postulated that usually higher total eDNA yield seems to correspond to higher detection probability (Piggott, 2016). However, there was no significant difference between the target DNA yields of centrifuged and filtered samples. Corresponding to this, the workflow that leads to the highest total DNA yield (DN4), was not the same workflow that lead to the highest target DNA yield (HS3). These differences could be due to species‐specific properties of the eDNA (Wang et al., 2013), but it could be also due to the nonhomogenous distribution of the target eDNA particles (Barnes & Turner, 2016). It should also be considered that filters had to be covered with the double amount of lysis buffer when using the high salt protocol and that only half of the mixture was used for extraction which lead to a loss of genetic material. Thus, it is consistent that the total DNA yield was lower in filter samples and it is remarkable that nevertheless, a filtration workflow (HS3) lead to the highest target DNA yield. We conclude that this workflow is particularly effective for the eDNA detection of *M. fossilis*.

In terms of eDNA extraction methods, the study of Piggott (2016) already demonstrated how important the choice of the eDNA extraction protocol is, as it has the strongest effects on detection probability alongside with choice of sampling and PCR strategy. In our study, we tested the DNeasy Blood & Tissue Kit (Qiagen) and a CTAB DNA extraction protocol, because they are commonly used in eDNA studies (Ficetola et al., 2008; Piggott, 2016; Thomsen, Kielgast, Iversen, Møller, et al., 2012; Thomsen, Kielgast, Iversen, Wiuf, et al., 2012; Turner et al., 2015). The high salt DNA extraction, which we tested in this study, was to the best of our knowledge not used in previous eDNA studies targeting *M. fossilis* or similar method papers. We revealed that there was no significant difference in total DNA yield between the high salt protocol and the DNeasy Blood & Tissue Kit (Figure 3). The CTAB method yielded significantly less total DNA than the two other methods (Figure 3). Furthermore, the target DNA yield of the high salt protocol was significantly higher than the yield of the CTAB method, while the DNeasy Kit showed no significant difference to both other methods. Most studies that compare DNA extraction methods tested commercial extraction Kits, for example, Qiagen DNeasy Blood & Tissue Kit, MoBio PowerWater DNA Isolation Kit, or phenol–chloroform–isoamyl alcohol extraction protocols (Deiner et al., 2015; Goldberg et al., 2011; Hinlo et al., 2017; Lin, Zhang, & Yao, 2019). We demonstrated that our high salt protocol leads to equal or higher total and target DNA yields in water samples than the DNeasy Blood & Tissue Kit and the phenol–chloroform DNA extraction (Figure 3). While the DNA extraction with the DNeasy Blood & Tissue Kit costs 3.48 € per sample and the CTAB protocol adds up to 0.3 € per sample, the high salt DNA extraction costs 0.1 € per sample and is thus more cost‐efficient. It is also advantageous that the ingredients for the high salt DNA extraction are nontoxic, unlike the ingredients of the phenol–chloroform DNA extraction. Overall, the high salt DNA extraction is a valid alternative to other DNA extraction methods. We furthermore emphasize with our results that also the combination of preservation, capture, and extraction methods has significant effects on total and target eDNA yields of water samples. Our results correspond well to the results of Deiner et al. (2015), who did not find a significant main effect of capture protocols alone on DNA yield but did find a highly significant effect of interaction between capture and extraction protocol. Additionally, Deiner et al. (2015) also found significant effects of extraction protocol on total eDNA yield, similar to the findings in our study.

### 4.2 Sediment samples

In our study, we tested six different eDNA workflows for sediment samples, based on workflows that were already applied in previous studies. For instance, we adapted the phosphate buffer pretreatment developed by Taberlet et al. (2012) in combination with the herein recommended NucleoSpin Soil extraction Kit (Figure 2). We also tested new method combinations such as the phosphate buffer pretreatment combined with the CTAB DNA extraction (Turner et al., 2015) and the high salt DNA extraction (Figure 2). Tab erlet et al. (2012) developed the phosphate buffer method for metabarcoding studies, because with this method, larger amounts of starting material can be used, which was supposed to better represent the local biodiversity. Despite that Taberlet et al. (2012) achieved sound results using the phosphate buffer pretreatment, in our study, the usage of phosphate buffer lead to significantly lower total and target DNA yields in sediment samples (Figure 4). Furthermore, phosphate buffer pretreatment resulted in much lower detection rates in combination with two out of three extraction methods. Thus, even though a larger amount of sediment (20 g) was used with the phosphate buffer pretreatment than with the nonpretreated samples (high salt and NucleoSpin = 500 mg; CTAB = 5 g), overall, the phosphate buffer treatment led to lower DNA yields and lower detection rates. It is known that soil and aqueous sediment contain large amounts of humic substances, which can inhibit PCR/ qPCR and thus lead to false‐negative results (Sidstedt et al., 2015). Even though we used an inhibitor removal kit for the sediment samples, the large amount of sediment (20 g) might have led to high concentrations of humic substances, so that the detection of *M. fossilis* in those samples was inhibited. Nevertheless, a 100% detection rate was obtained by combining the phosphate buffer pretreatment with the NucleoSpin Soil Kit (N1, Figure 5). An explanation could be that the NucleoSpin Soil Kit did remove humic substances more efficiently than the other two extraction methods. The sediment extraction protocol of Turner et al. (2015) was performed using 5 g sediment, preserved in 10 ml CTAB buffer and conducting a CTAB DNA extraction (C2). This workflow led to a detection rate of 87.5%, while the workflows H2 and N2, which only used 500 mg sediment, led to a 100% detection rate. This could also imply that smaller sediment amounts lead to less inhibition.

The significantly highest total and target DNA yield was obtained using workflow H2, a standard high salt DNA extraction of 500 mg sediment without pretreatment (Figure 4). This protocol also achieved a 100% detection rate and is the recommendable procedure for future eDNA studies of *M. fossilis* targeting sediment (Figure 5). The comparative water sample (1.5 L, filtered, extracted with high salt), that was taken directly before sediment sampling, showed a *C*_t_ value of 33.95, while the mean *C*_t_ value of the best sediment workflow was 33.06. Thus, the target DNA yield of the 500 mg sediment samples was higher than the target DNA yield of the 1.5 L water sample. This result could indicate that eDNA of *M. fossilis* is more concentrated in sediment than in water, supporting the findings of Turner et al. (2015). However, we cannot statistically support that hypothesis as we took only one comparative water sample. We emphasize that sediment samples can be used for the monitoring of *M. fossilis* and that the choice of workflows significantly influences total and target DNA yield as well as the detection rate.

### 4.3 Seasonal eDNA sampling

Our third experiment was the analysis of monthly water samples at an agricultural ditch. The *M. fossilis* population was confirmed by using fish traps (Table S8). By analyzing the total DNA yield of the monthly samples, we were able to show that the total DNA amount in the ditch varied strongly over the year. We furthermore demonstrate that in the summer months from June to September, the total DNA was high, while it was low from November, February, and March (Figure 6). The target DNA yield of *M. fossilis* showed an almost similar trend to the total DNA yield, as it was high in the summer months from June to September and low in the months of November, February, and March (Figure 6). However, in April and May, total and target DNA showed opposing trends.

Former studies have shown that eDNA monitoring is a valid tool for reflecting seasonal changes in species abundance and community composition in aquatic habitats (Bista et al., 2017; Sigsgaard et al., 2017). Additionally, eDNA amount of a species can increase during its breeding period (Spear, Groves, Williams, & Waits, 2015). As *M. fossilis* usually breeds from April to June, depending on the water temperature, the increased target DNA yield starting from May could have reflected that. However, the target DNA yield was even higher in September, which is not a breeding month for *M. fossilis*. Yamanaka and Minamoto (2016) presented eDNA monitoring as a valid tool to track the seasonal migration of fish populations. They described that after the emigration of a fish population from a summer habitat, eDNA was not detectable at the same habitat during the winter months. In the case of *M. fossilis*, there are no seasonal migration patterns known up to today, although we cannot exclude that they periodically change their location. However, it seems to be more likely that the increased target DNA yield reflects the activity and metabolism of the fish. Klymus et al. (2015) have shown that eDNA shedding is diet‐dependent and that more nourishment leads to more excretion and thus also to a higher eDNA shedding rate, which might be related to higher nourishment (e.g., insect larvae or snails) in the summer and spring months. However, we can only speculate about the reasons for the increased total and target DNA yields in summer, as the samples were taken from a natural system that is influenced by multiple factors. Nevertheless, our findings indicate that sampling from June to September could lead to better results for the monitoring of *M. fossilis*.

## 5 CONCLUSION

Our study has shown that water sample preservation with ethanol and sodium acetate, used in former eDNA studies, impaired total and target DNA yield as well as the detection rates when samples were stored for two weeks or longer. Based on that, we recommend freezing water samples at −20°C for long‐term storage instead of chemical fixation. Our results show that an inexpensive high salt DNA extraction achieved high total and target DNA yields for water and sediment samples and thus can be a valid alternative to commercial DNA extraction kits. We demonstrated that even small amounts of sediment achieved high total and target DNA yields in combination with the high salt DNA extraction. According to our results, the target eDNA concentration is higher in sediment than in water, which shows that sediment samples are recommendable for the monitoring of *M. fossilis*. We showed that that in a natural habitat of *M. fossilis,* total and target DNA concentration was highest in summer months from June to September. Hence, we recommend this period for sample collection in future eDNA surveys of *M. fossilis*.

## CONFLICT OF INTEREST

The authors declare that there is no conflict of interest.

## AUTHOR CONTRIBUTIONS

**Lena Maureen Kusanke:** Conceptualization (lead); Data curation (lead); Formal analysis (lead); Investigation (lead); Methodology (lead); Project administration (equal); Software (lead); Visualization (lead); Writing‐original draft (lead); Writing‐review & editing (lead). **Jörn Panteleit:** Methodology (equal); Writing‐review & editing (supporting). **Stefan Stoll:** Conceptualization (equal); Funding acquisition (equal); Project administration (equal); Supervision (equal); Writing‐review & editing (supporting). **Egbert Korte:** Investigation (supporting); Resources (supporting). **Eike Sünger:** Investigation (supporting); Resources (supporting). **Ralf Schulz:** Conceptualization (supporting); Funding acquisition (equal); Project administration (lead); Resources (equal); Supervision (equal); Writing‐review & editing (supporting). **Kathrin Theissinger:** Conceptualization (equal); Methodology (equal); Supervision (lead); Validation (lead); Writing‐original draft (supporting); Writing‐review & editing (supporting).

## Supporting information

Appendix S1

Appendix S2

Figure S1

Figure S2

Figure S3

Figure S4

Figure S5

## Data Availability

The data that were generated during this study are published on Dryad: https://doi.org/10.5061/dryad.4xgxd256h.
