# Supplementary material for: Detection of the endangered European weather loach (Misgurnus fossilis) via water and sediment samples: Testing multiple eDNA workflows
Source: Ecol Evol. 2020 Jul 6;10(15):8331–44. doi: 10.1002/ece3.6540 (PMC7417210; doi:10.1002/ece3.6540)
Supplement: Supplementary file 1 — Appendix S1 [file ECE3-10-8331-s001.docx]

# Appendix S1

**Table S1** P-values calculated using the pairwise Wilcoxon rank sum test comparing the total DNA yield in water samples of the twelve tested workflows.

|  | CB1 | CB2 | CB3 | CB4 | DN1 | DN2 | DN3 | DN4 | HS1 | HS2 | HS3 |
| --- | --- | --- | --- | --- | --- | --- | --- | --- | --- | --- | --- |
| CB2 | 0.71299 | - | - | - | - | - | - | - | - | - | - |
| CB3 | 0.01298 | 0.02574 | - | - | - | - | - | - | - | - | - |
| CB4 | 0.00150 | 0.00031 | 0.65531 | - | - | - | - | - | - | - | - |
| DN1 | 0.00283 | 0.00628 | 0.25372 | 0.00415 | - | - | - | - | - | - | - |
| DN2 | 0.00150 | 0.00031 | 0.65531 | 0.17658 | 0.02574 | - | - | - | - | - | - |
| DN3 | 0.00150 | 0.00031 | 0.03438 | 0.00628 | 0.00031 | 0.00060 | - | - | - | - | - |
| DN4 | 0.00150 | 0.00031 | 0.00031 | 0.00031 | 0.00031 | 0.00031 | 0.00031 | - | - | - | - |
| HS1 | 0.33322 | 0.29661 | 0.00280 | 0.00031 | 0.00031 | 0.00031 | 0.00031 | 0.00031 | - | - | - |
| HS2 | 0.00706 | 0.09443 | 0.11734 | 0.00031 | 0.04390 | 0.00031 | 0.00031 | 0.00031 | 0.00167 | - | - |
| HS3 | 0.00150 | 0.00031 | 0.00031 | 0.00031 | 0.00031 | 0.00031 | 0.00167 | 0.00415 | 0.00031 | 0.00031 | - |
| HS4 | 0.00150 | 0.00031 | 0.00031 | 0.00031 | 0.00031 | 0.00031 | 0.00117 | 0.04390 | 0.00031 | 0.00031 | 0.04390 |

**Table S2** Mean absorbance and mean ratio of absorbance at 260nm and 280nm and the standard deviation measured in DNA extracts of water samples using the Nanodrop Spectrophotometer ND 1000.

| Workflow | A260 | SD A260 | A280 | SD A280 | 260/280 | SD 260/280 |
| --- | --- | --- | --- | --- | --- | --- |
| CB1 | 0.014 | 0.004 | 0.008 | 0.005 | 1.155 | 1.604 |
| CB2 | 0.016 | 0.006 | 0.010 | 0.013 | 2.030 | 2.520 |
| CB3 | 0.058 | 0.041 | 0.023 | 0.023 | 1.933 | 1.237 |
| CB4 | 0.060 | 0.023 | 0.035 | 0.017 | 2.459 | 1.458 |
| DN1 | 0.029 | 0.009 | 0.028 | 0.010 | 0.990 | 0.422 |
| DN2 | 0.043 | 0.008 | 0.016 | 0.005 | 2.513 | 1.474 |
| DN3 | 0.152 | 0.008 | 0.106 | 0.005 | 1.565 | 1.474 |
| DN4 | 0.788 | 0.213 | 0.550 | 0.156 | 1.443 | 0.037 |
| HS1 | 0.012 | 0.004 | 0.008 | 0.005 | 1.344 | 1.481 |
| HS2 | 0.021 | 0.004 | 0.013 | 0.007 | 1.754 | 2.986 |
| HS3 | 0.405 | 0.095 | 0.290 | 0.069 | 1.401 | 0.019 |
| HS4 | 0.524 | 0.207 | 0.395 | 0.156 | 1.332 | 0.419 |

**Table S3** P-values calculated with the pairwise Wilcoxon rank sum test for the total DNA yield of sediment samples.

|  | C1 | C2 | H1 | H2 | N1 |
| --- | --- | --- | --- | --- | --- |
| C2 | 0.13969 | - | - | - | - |
| H1 | 0.00026 | 0.00636 | - | - | - |
| H2 | 0.00026 | 0.00026 | 0.00026 | - | - |
| N1 | 0.00280 | 0.38228 | 0.00026 | 0.00026 | - |
| N2 | 0.04375 | 0.02584 | 0.00026 | 0.00026 | 0.00026 |

**Table S4** Mean absorbance and mean ratio of absorbance at 260nm and 280nm and the standard deviation measured in DNA extracts of sediment samples using the Nanodrop Spectrophotometer ND 1000.

| Workflow | A260 | SD A260 | A280 | SD A280 | 260/280 | SD 260/280 |
| --- | --- | --- | --- | --- | --- | --- |
| C1 | 0.564 | 0.142 | 0.342 | 0.086 | 1.651 | 0.033 |
| C2 | 0.381 | 0.244 | 0.205 | 0.135 | 2.164 | 0.660 |
| H1 | 0.045 | 0.013 | 0.023 | 0.009 | 2.199 | 0.840 |
| H2 | 1.358 | 0.188 | 0.745 | 0.112 | 1.829 | 0.028 |
| N1 | 0.281 | 0.048 | 0.174 | 0.031 | 1.656 | 0.112 |
| N2 | 0.717 | 0.071 | 0.429 | 0.043 | 1.679 | 0.035 |

**Table S5** P-values calculated using the pairwise Wilcoxon rank sum test for Ct values of water samples; P-Values <0.05 indicate significant differences between two workflows.

|  | CB1 | CB2 | CB3 | CB4 | DN1 | DN2 | DN3 | DN4 | HS1 | HS2 | HS3 |
| --- | --- | --- | --- | --- | --- | --- | --- | --- | --- | --- | --- |
| CB2 | 0.18491 | - | - | - | - | - | - | - | - | - | - |
| CB3 | 0.00087 | 0.00068 | - | - | - | - | - | - | - | - | - |
| CB4 | 0.00087 | 0.00068 | 0.81073 | - | - | - | - | - | - | - | - |
| DN1 | 0.00179 | 0.00068 | 0.00035 | 0.00035 | - | - | - | - | - | - | - |
| DN2 | 0.00087 | 0.00068 | 0.00035 | 0.00035 | 0.00068 | - | - | - | - | - | - |
| DN3 | 0.00087 | 0.00068 | 0.47031 | 1.00000 | 0.00035 | 0.00035 | - | - | - | - | - |
| DN4 | 0.00087 | 0.00068 | 0.00035 | 0.00531 | 0.00035 | 0.00035 | 0.00035 | - | - | - | - |
| HS1 | 0.00087 | 0.00068 | 0.00035 | 0.00035 | 0.52943 | 0.00531 | 0.00035 | 0.00035 | - | - | - |
| HS2 | 0.00087 | 0.00068 | 0.00087 | 0.00035 | 0.00035 | 0.00135 | 0.00035 | 0.00035 | 0.00035 | - | - |
| HS3 | 0.00087 | 0.00068 | 0.00087 | 0.00087 | 0.00035 | 0.00035 | 0.00035 | 0.01165 | 0.00035 | 0.00035 | - |
| HS4 | 0.00087 | 0.00068 | 0.00531 | 0.00224 | 0.00035 | 0.00035 | 0.00035 | 0.59167 | 0.00035 | 0.00035 | 0.02285 |

**Table S6** P-values calculated with the pairwise Wilcoxon rank sum test for the Ct values of sediment samples.

|  | C1 | C2 | H1 | H2 | N1 |
| --- | --- | --- | --- | --- | --- |
| C2 | 0.0123 | - | - | - | - |
| H1 | 0.8400 | 0.0123 | - | - | - |
| H2 | 0.0017 | 0.0049 | 0.0017 | - | - |
| N1 | 0.0017 | 0.0624 | 0.0017 | 0.0012 | - |
| N2 | 0.0017 | 0.1504 | 0.0017 | 0.0012 | 0.9591 |

**Table S7** Mean absorbance and mean ratio of absorbance at 260nm and 280nm and the standard deviation measured in DNA extracts of samples from the ditch in Rheinzabern, Germany (49.09324, 8.30060) using the Nanodrop Spectrophotometer ND 1000.

| Samples | A260 | SD A260 | A280 | SD A280 | 260/280 | SD 260/280 |
| --- | --- | --- | --- | --- | --- | --- |
| Feb | 0.59 | 0.02 | 0.39 | 0.03 | 1.52 | 0.05 |
| Mar | 0.52 | 0.05 | 0.28 | 0.03 | 1.84 | 0.06 |
| Apr | 1.79 | 0.25 | 0.94 | 0.14 | 1.91 | 0.03 |
| May | 0.54 | 0.02 | 0.29 | 0.01 | 1.85 | 0.04 |
| Jun | 4.32 | 0.02 | 2.28 | 0.01 | 1.89 | 0.00 |
| Jul | 3.31 | 0.64 | 1.74 | 0.35 | 1.90 | 0.02 |
| Aug | 4.23 | 0.89 | 2.16 | 0.46 | 1.96 | 0.01 |
| Sep | 3.98 | 0.71 | 2.13 | 0.38 | 1.87 | 0.01 |
| Oct | 1.77 | 0.02 | 0.94 | 0.02 | 1.89 | 0.01 |
| Nov | 0.16 | 0.04 | 0.10 | 0.03 | 1.60 | 0.10 |

**Table S8** Number, size and gender of M. fossilis caught in fish traps at the studied ditch in Rheinzabern, Germany (49.09324, 8.30060) in April 2018.

| Date | Length | Number of Individuals | Sex |
| --- | --- | --- | --- |
| *13.04.2018* | 17,0 | 2 | Male |
| *13.04.2018* | 17,0 | 1 | Female |
| *13.04.2018* | 18,0 | 8 | Male |
| *13.04.2018* | 18,0 | 1 | Female |
| *13.04.2018* | 19,0 | 3 | Male |
| *13.04.2018* | 21,0 | 1 | Female |
| *13.04.2018* | 22,0 | 1 | Male |
| *13.04.2018* | 24,0 | 2 | Female |
| *13.04.2018* | 25,0 | 1 | Female |

**Fig. S1** Comparison of (A) the total DNA yield and (B) the Ct values of the three DNA extraction protocols for water samples. Ct values are inversely proportional to the target DNA amount of a sample, thus the lower the Ct value, the higher is the target DNA amount.

**Fig. S2** Comparison of (A) the total DNA yield and (B) the Ct values of sample preservation methods in water samples. Ct values are inversely proportional to the target DNA amount of a sample, thus the lower the Ct value, the higher is the target DNA amount.

**Fig. S3** Comparison of (A) the total DNA yield and (B) the Ct values of DNA capture methods in water samples. Ct values are inversely proportional to the target DNA amount of a sample, thus the lower the Ct value, the higher is the target DNA amount.

**Fig. S4** Comparison of (A) the total DNA yield and (B) the Ct values of sediment samples with and without pretreatment. Ct values are inversely proportional to the target DNA amount of a sample, thus the lower the Ct value, the higher is the target DNA amount.

**Fig. S5** Comparison of (A) the total DNA yield and (B) the Ct values of the three DNA extraction protocols for sediment samples. Ct values are inversely proportional to the target DNA amount of a sample, thus the lower the Ct value, the higher is the target DNA amount.
