# Supplementary material for: Detection of the endangered European weather loach (Misgurnus fossilis) via water and sediment samples: Testing multiple eDNA workflows
Source: Ecol Evol. 2020 Jul 6;10(15):8331–44. doi: 10.1002/ece3.6540 (PMC7417210; doi:10.1002/ece3.6540)
Supplement: Supplementary file 2 — Appendix S2 [file ECE3-10-8331-s002.docx]

Appendix S2

# **High Salt DNA Extraction Protocol**

## **Centrifuged Samples**

First, 450 µL stain extraction buffer (SEB) buffer, 100 µL 10% SDS and 5 µL proteinase K (10 mg/mL) was added to the organic pellet. Subsequently, the samples were incubated for 1 h at 60°C and they were vortexed every 10 min. After the incubation, 555 µL of this mixture were transferred into a new microcentrifuge tube, vortexed and briefly centrifuged. Then, we added 350 µL of 5 M NaCl. The mixture was vortexed and centrifuged for 30 min at 16,200 x g. In the following, 600 µL supernatant was transferred into a new tube and 600 µL of ice-cold Isopropanol was added. The tubes were reversed a few times to mix. Then centrifugation was carried out for 10 min at 4°C at 16,200 x g. After the centrifugation, the supernatant was discarded, and the tubes were dried with tissue paper. Then 200 µL ice-cold 70% Ethanol was added and another centrifugation for 10 min at 4°C at 16,200 x g was carried out. The supernatant was then removed by pouring it off and a pipette was used to remove droplets. Then the pellet was dried at 60°C for 5 to 20 min until all EtOH had evaporated. The pellet was dissolved in 100 µL of H_2_O.

## **Filter Samples**

For filter samples, 900 µL SEB buffer, 200 µL 10% SDS and 10 µL proteinase K were used to cover the filters completely in liquid during the incubation. The filters were cut into small pieces before the liquid was added. Subsequently, the samples were incubated for 1 h at 60°C and they were vortexed every 10 min. After the incubation, 555 µL of this mixture were transferred into a new microcentrifuge tube, vortexed and briefly centrifuged. Then, we added 350 µL of 5 M NaCl. The mixture was vortexed and centrifuged for 30 min at 16,200 x g. In the following, 600 µL supernatant was transferred into a new tube and 600 µL of ice-cold Isopropanol was added. The tubes were reversed a few times to mix. Then centrifugation was carried out for 10 min at 4°C at 16,200 x g. After the centrifugation, the supernatant was discarded, and the tubes were dried with tissue paper. Then 200 µL ice-cold 70% Ethanol was added and another centrifugation for 10 min at 4°C at 16,200 x g was carried out. The supernatant was then removed by pouring it off and a pipette was used to remove droplets. Then the pellet was dried at 60°C for 5 to 20 min until all EtOH had evaporated. The pellet was dissolved in 100 µL of H_2_O.
