# Supplementary figures and images for: Detection of the endangered European weather loach (Misgurnus fossilis) via water and sediment samples: Testing multiple eDNA workflows
Source: Ecol Evol. 2020 Jul 6;10(15):8331–44. doi: 10.1002/ece3.6540 (PMC7417210; doi:10.1002/ece3.6540)

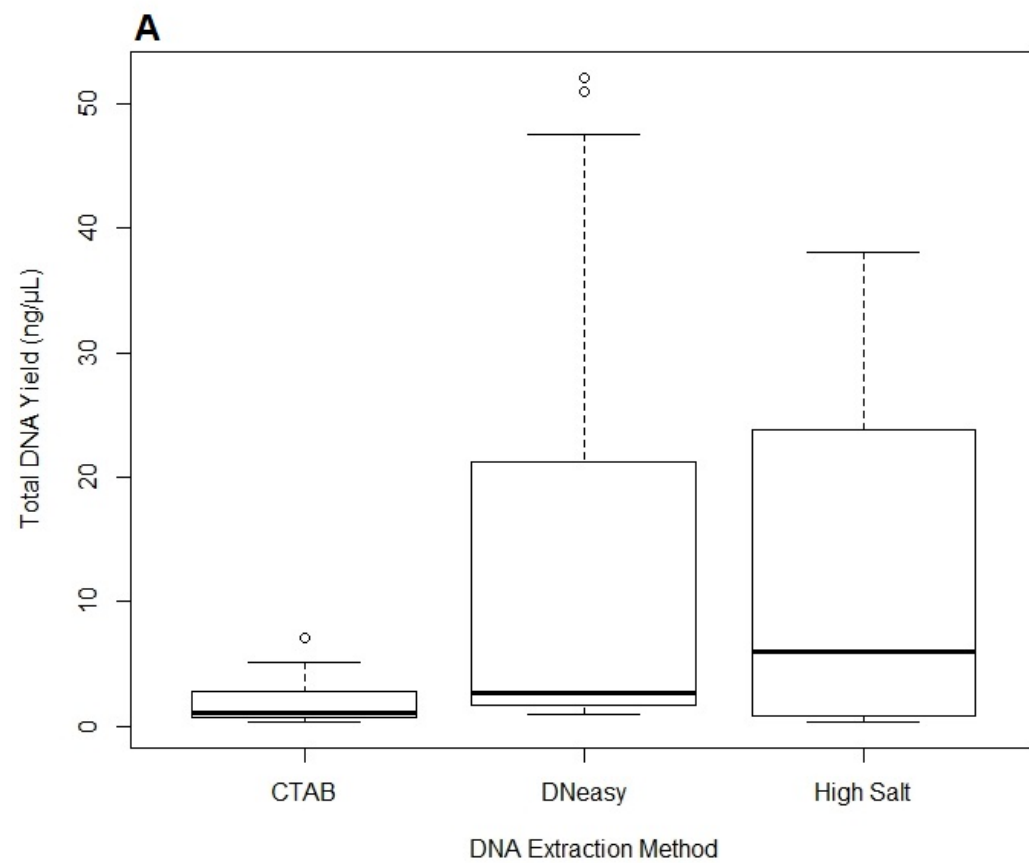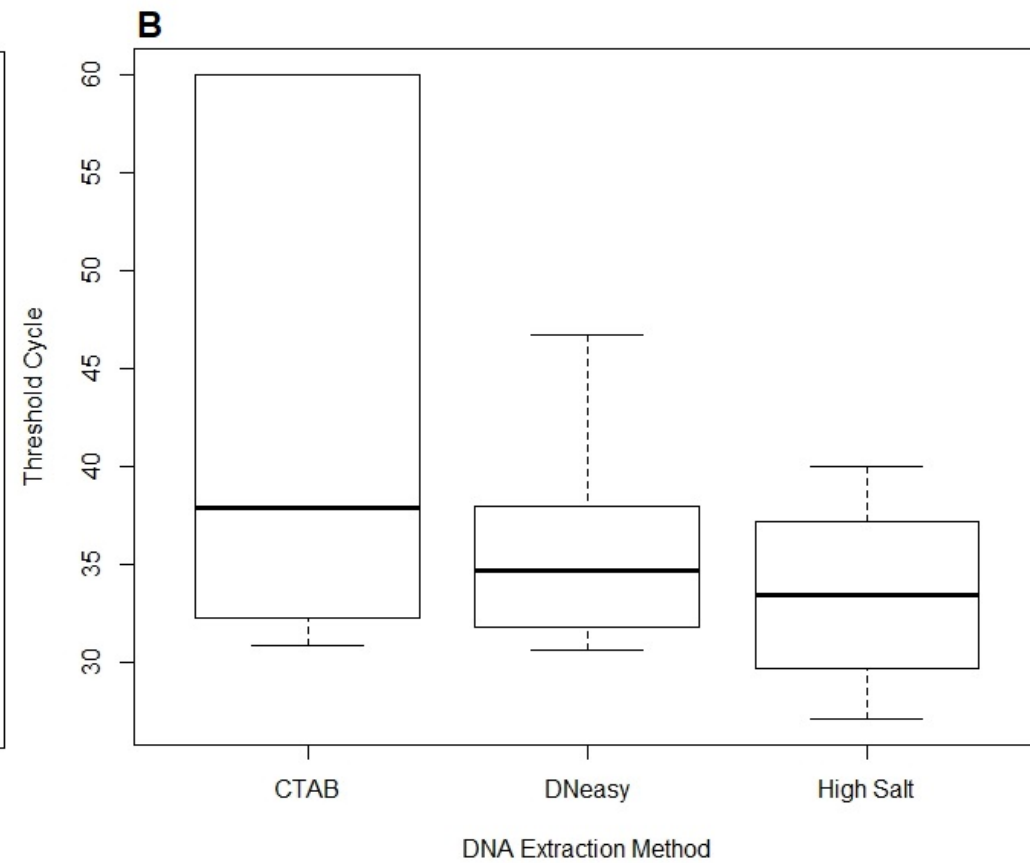

Supplement: Supplementary file 3 — Figure S1 [file ECE3-10-8331-s003.pdf]

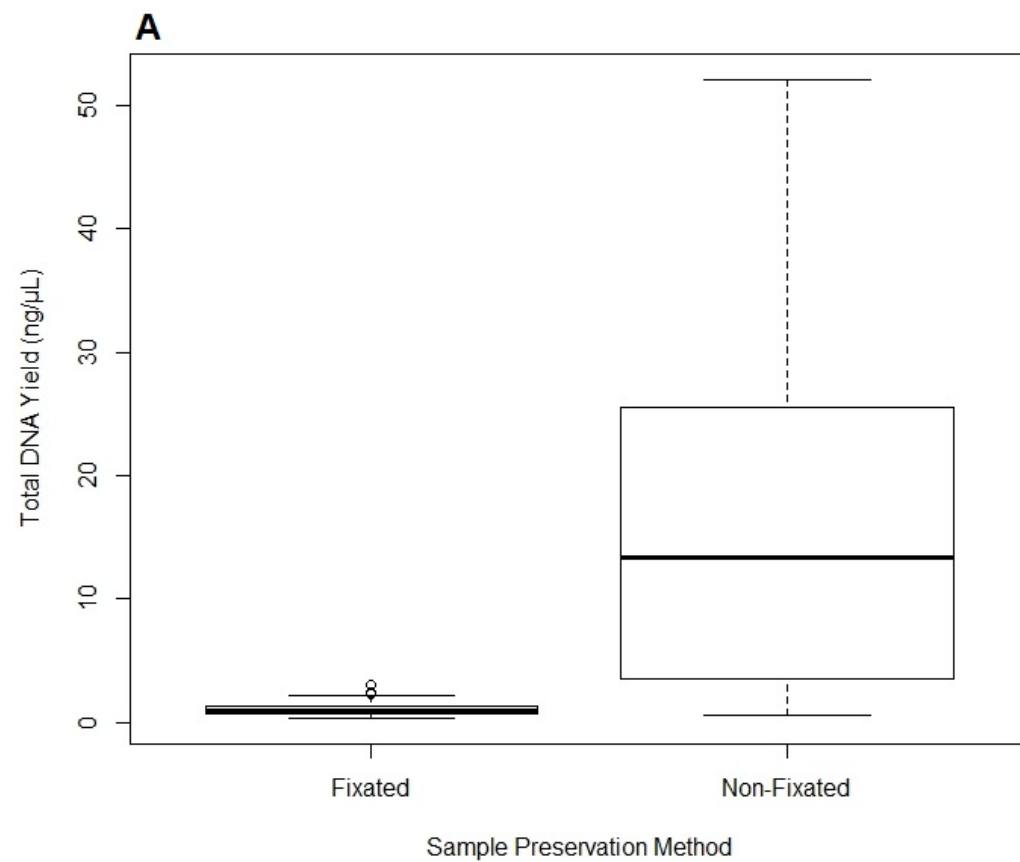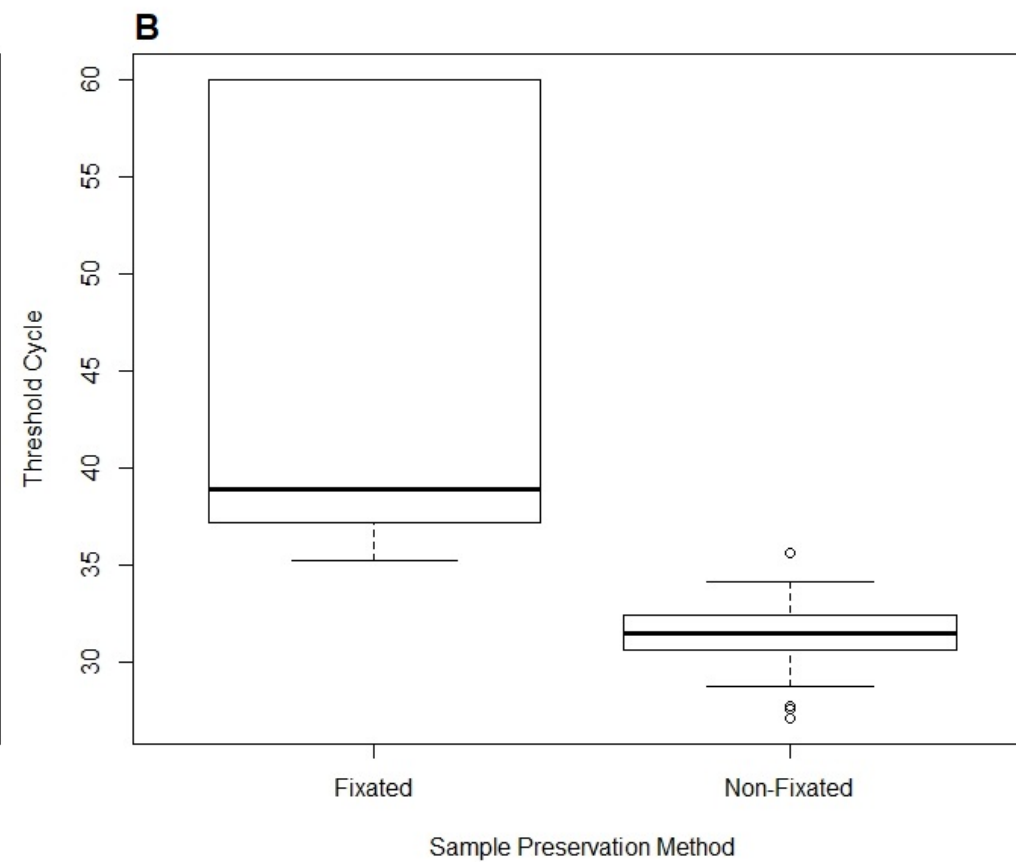

Supplement: Supplementary file 4 — Figure S2 [file ECE3-10-8331-s004.pdf]

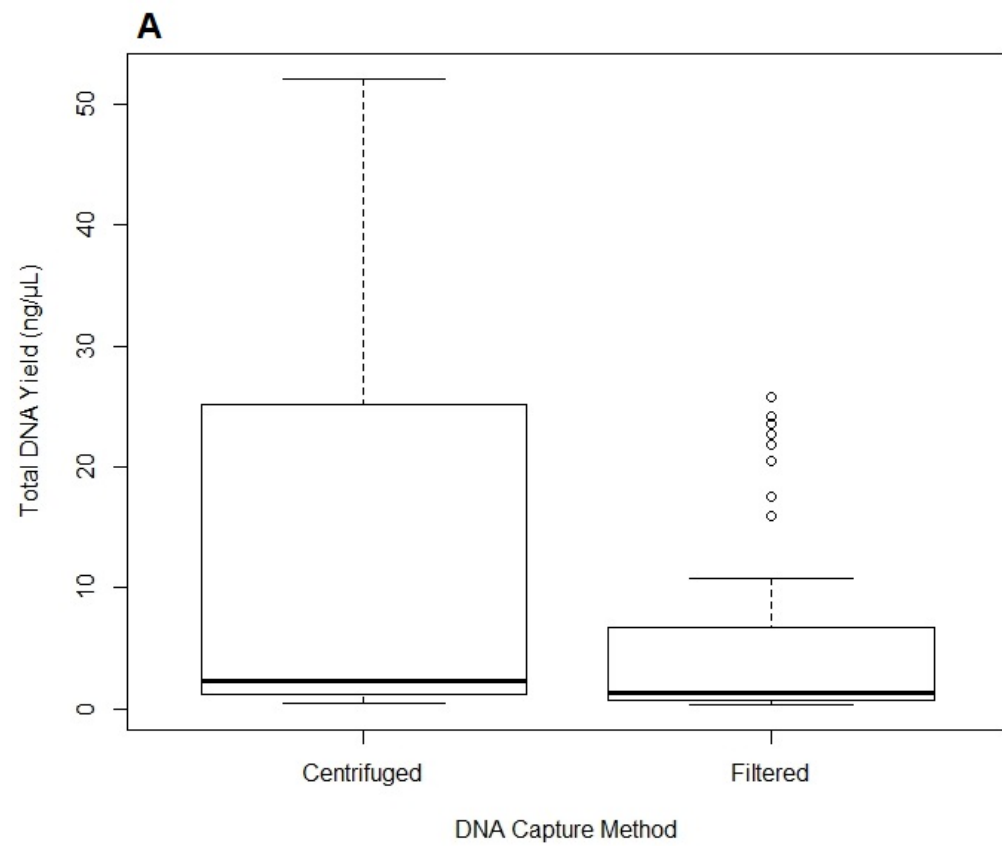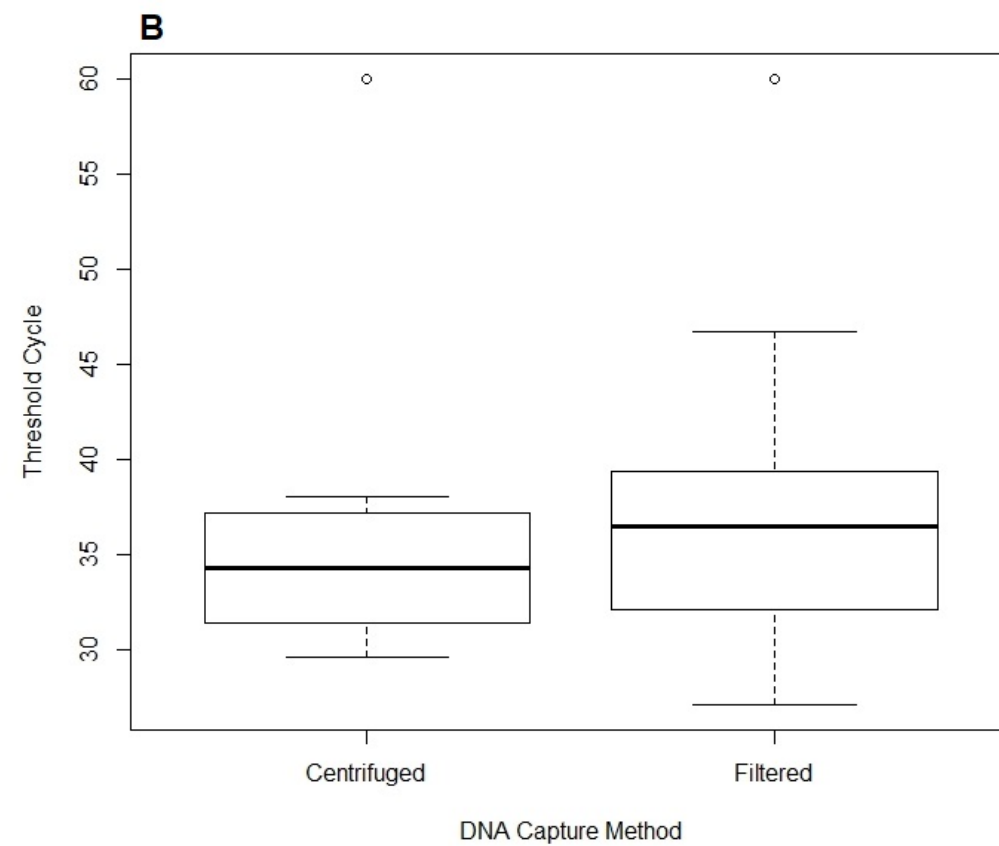

Supplement: Supplementary file 5 — Figure S3 [file ECE3-10-8331-s005.pdf]

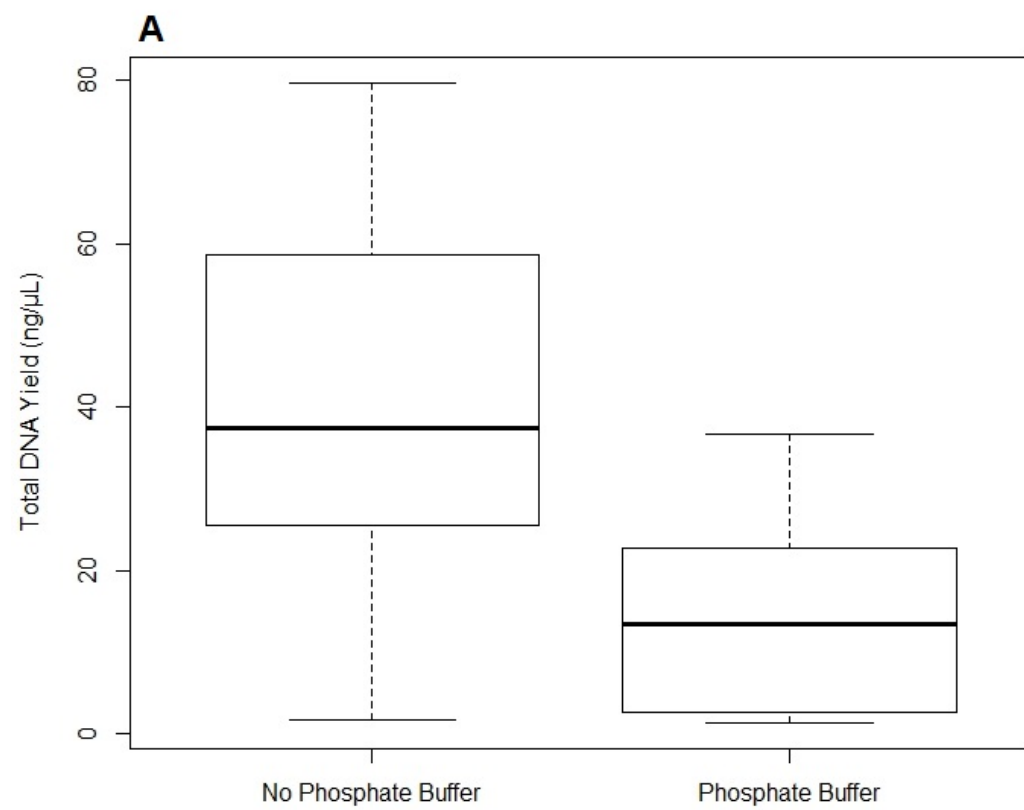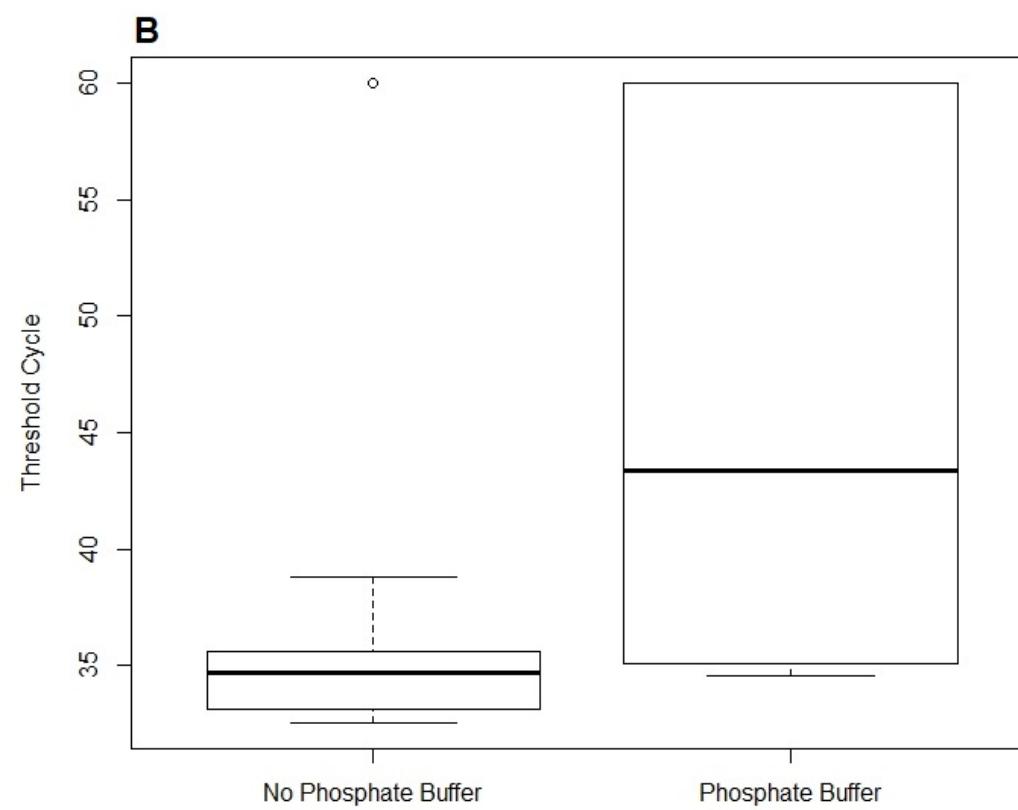

Supplement: Supplementary file 6 — Figure S4 [file ECE3-10-8331-s006.pdf]

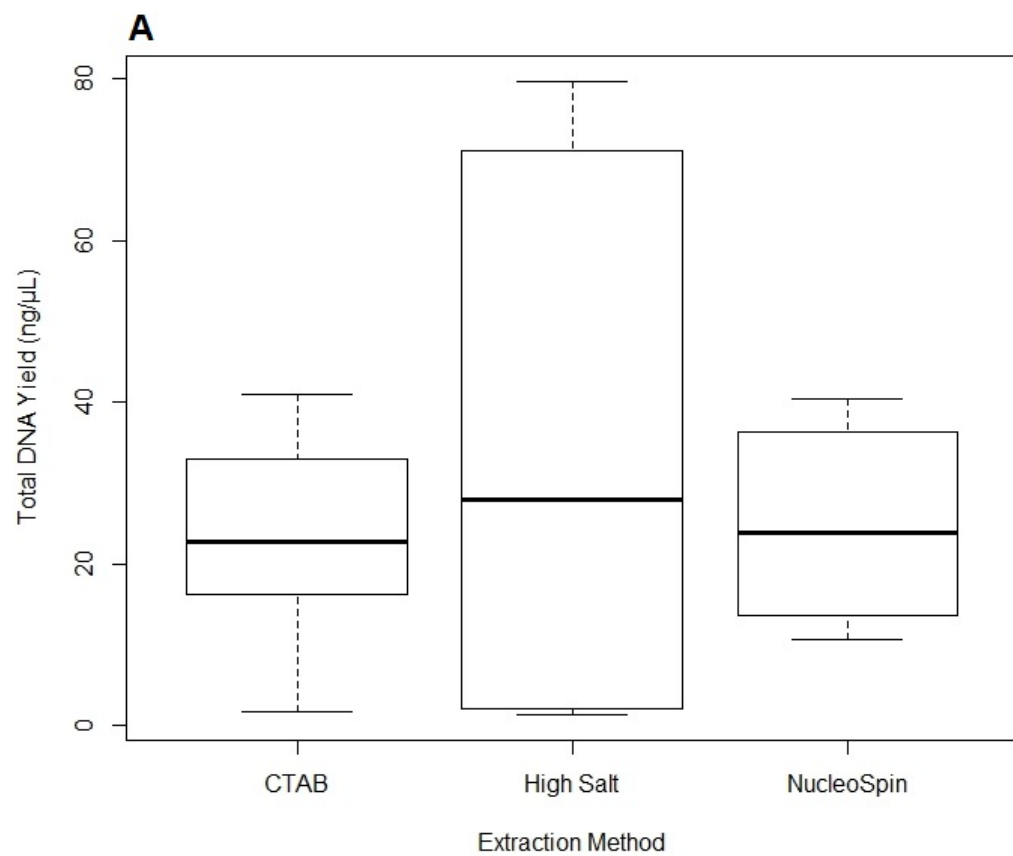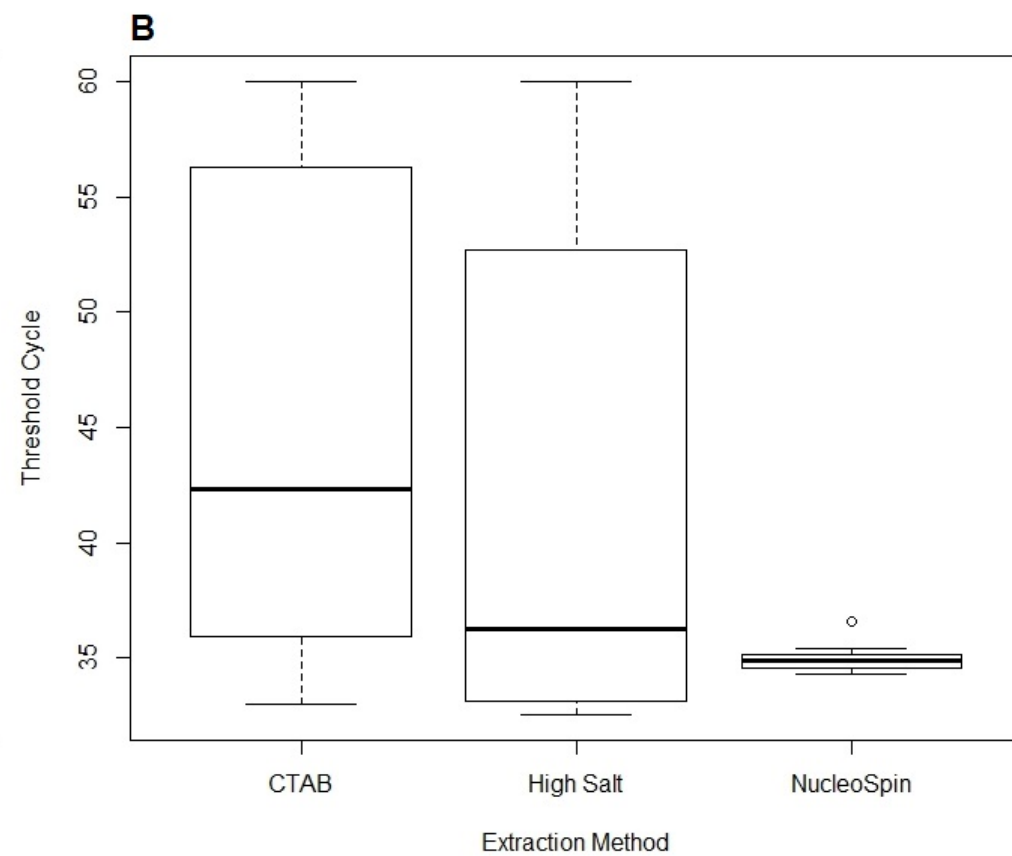

Supplement: Supplementary file 7 — Figure S5 [file ECE3-10-8331-s007.pdf]
